# Epidemiology of Cardiac Myxoma in the Kingdom of Bahrain

**DOI:** 10.7759/cureus.55704

**Published:** 2024-03-07

**Authors:** Tarique S Chachar, Nooraldaem Yousif, Husam A Noor, Dayaram Makwana, Mohamed K Alkhayat, Habib Tareif, Zaid R Arekat, Haitham Amin

**Affiliations:** 1 Cardiology, Mohammed bin Khalifa bin Salman Al Khalifa Specialist Cardiac Centre, Awali, BHR; 2 Interventional Cardiology, Mohammed bin Khalifa bin Salman Al Khalifa Specialist Cardiac Centre, Awali, BHR; 3 Cardiothoracic Surgery, Mohammed bin Khalifa bin Salman Al Khalifa Specialist Cardiac Centre, Awali, BHR

**Keywords:** left atrial myxoma, cardiac tumors, embolism, ventricular septum, benign tumors, right atrial myxoma, cardiac masses

## Abstract

Background: Cardiac myxomas (CM) are the most prevalent type of primary cardiac tumour. The majority of primary cardiac tumours, including CM, are found to be benign. In the context of this study, the objective was to investigate and analyse the experience of CM over a period of 10 years, specifically in Bahrain. By examining this particular subset of cardiac tumours, valuable insights can be gained regarding their prevalence, clinical presentation, diagnostic methods, treatment approaches, and outcomes in the Bahraini population.

Methods: We retrospectively evaluated the medical records of 20 patients who presented with CM at the Mohammed bin Khalifa bin Salman Al Khalifa Specialist Cardiac Centre in the Kingdom of Bahrain from January 2010 to January 2021. All patients had transthoracic echocardiography to establish a preoperative diagnosis. All of the patients underwent an operation using the median sternotomy, and a histopathology examination confirmed the final diagnosis.

Results: The mean age at the time of presentation was 57 (± 18.1) years, ranging from 17 to 80 years, and 55% (12 patients) were female. Dyspnea (n=8, 40%) and peripheral embolism (n=4, 20%), which include cerebrovascular accidents and acute monocular vision loss, were the most frequently observed symptoms. The largest diameter of the myxoma was 5.1 cm (±1.7). The left atrium was the predominant location for myxoma formation (n=16, 80%), with the majority of the myxomas attached to the atrial septum.

Conclusion: CM poses a significant risk of cardiac and systemic complications. Early detection and timely gross-complete resection result in excellent early and long-term outcomes.

## Introduction

Cardiac myxoma (CM) is the most prevalent kind of primary cardiac neoplasm, accounting for 30-50% of all primary heart tumours, with a 0.5 per million population yearly incidence [1]. Atrial myxomas occur mostly in the third to sixth decades and are predominantly female at 2:1 [2]. CM can be smooth, round, or gelatinous in appearance, or friable and irregular, i.e., polypoid or papillary. They frequently adhere to a sessile or pedunculated base and may contain a hemorrhagic core. In a presentation, the diameter is typically 4-8 cm, and the mass is typically 150-180 g. These tumours arise from multipotent mesenchymal cells in the subendocardial space [3].

CM primarily develops in the left atrium in 70% of instances, the right atrium in 18% of cases, and both atria in fewer than 5% of cases. It can also originate from the ventricles in 3-4% of cases [4]. Atrial myxoma symptoms can range from obstructive to embolic, with a variety of arrhythmias and constitutional symptoms such as fever, malaise, anorexia, arthralgia, and weight loss. About 10% of cases are asymptomatic [5].

Atrial myxomas can appear as a component of the carney complex on rare occasions. They exhibit unusual skin pigmentation and frequently develop tumours in endocrine tissues such as the adrenal glands, thyroid, testes, and ovaries. Despite resections, patients with Carney's complex frequently develop recurrent atrial myxomas [6]. Surgical resection is the primary treatment for CM. The aim of this study was to perform an epidemiological analysis of CM over the past 10 years in the Kingdom of Bahrain.

## Materials and methods

This was a retrospective analysis of the medical records of 20 patients who were either admitted or referred from other hospitals to the Mohammed bin Khalifa bin Salman Al Khalifa Specialist Cardiac Centre in the Kingdom of Bahrain between January 2010 and January 2021. The cardiac centre is the sole facility of its kind in the kingdom and specialises in treating CM. Approval was obtained from the Medical Ethics Committee of the Mohammed bin Khalifa bin Sulman Al-Khalifa Specialist Cardiac Centre in Bahrain (approval number: CTD-RES-2024-006). It's worth noting that, due to the retrospective nature of the study, informed consent forms were exempted from review by the ethics committee. All patients' medical records and diagnostic tests were reviewed. Telephone calls to patients were used to collect some follow-up data. The study adhered to the principles outlined in the Declaration of Helsinki.

We analysed baseline demographic data from medical records, including age, sex, cardiovascular risk factors, prior history of stroke or other systemic embolic events, and family history of CM. Transthoracic echocardiography (TTE) was performed routinely on all patients following admission, and subsequently transesophageal echocardiography (TEE). Experienced cardiologists conducted all recordings and interpretations. The operative features and clinical outcomes were evaluated.

Diagnosis

TTE was performed routinely on all patients following admission using a Vivid E 95 cardiovascular ultrasound system (GE HealthCare Technologies, Inc., Chicago, Illinois, United States) and an EPIQ 7 system (Koninklijke Philips N.V., Amsterdam, Netherlands). TEE was performed for each patient with EPIC 7 (Koninklijke Philips N.V.). We used two-dimensional or parasternal M-mode images to measure the left atrial diameter (LAD), the left ventricular end-diastolic dimension (LVEDD), the left ventricular end-systolic dimension (LVESD), and the left ventricular ejection fraction (LVEF). We carefully examined the morphologic characteristics of CA. The tumour size was determined using the width and height of the attachment site. The myxomas were grouped by their shape (round, ovoid, or prolapsing), the presence of pedicles (narrow stalk vs. broad base), the amount of calcification and necrosis, and the type of surface irregularity (smooth vs. polyp). Planimetry of the mitral orifice was used to assess mitral stenosis and colour flow according to the guidelines. Doppler mapping was used to quantify the mitral valve's regurgitant jet area [7]. Multiple perspectives were used to evaluate these measurements. Experienced cardiologists made all recordings and interpretations.

## Results

Table 1 provides an overview of the patient population, including their mean age, gender distribution, and the most common symptoms observed.

**Table 1:** Demographic, clinical, and pathological variables (N=20)

Variables	Values
Male, n (%)	9 (45%)
Age (years), mean ± SD	57 ± 18.1
Medical History	
Diabetes mellitus, n (%)	8 (40%)
Systemic hypertension, n (%)	6 (30%)
Dyslipidaemia, n (%)	4 (20%)
Coronary artery disease, n (%)	3 (15%)
Smoking, n (%)	1 (5%)
Peripheral artery disease, n (%)	0
Chronic kidney disease no (%)	0
Stroke, n (%)	2 (10%)
Chronic obstructive pulmonary disease, n (%)	1 (5%)
Presentation	
Dyspnoea, n (%)	8 (40%)
Syncope, n (%)	1 (5%)
Palpitations, n (%)	1(5%)
Chest pain, n (%)	2 (10%)
Fever, n (%)	1 (5%)
Acute coronary syndrome, n (%)	1 (5%)
Stroke, n (%)	2 (10%)
Acute monocular vision loss, n (%)	2 (10%)
Arrhythmias, n (%)	1 (5%)
Tumor Characteristics	
Size largest diameter (cm), mean ± SD	5.1 ± 1.7
Location	
Left atrium, n (%)	16 (80%)
Right atrium, n (%)	4 (20%)

**Figure 1:**
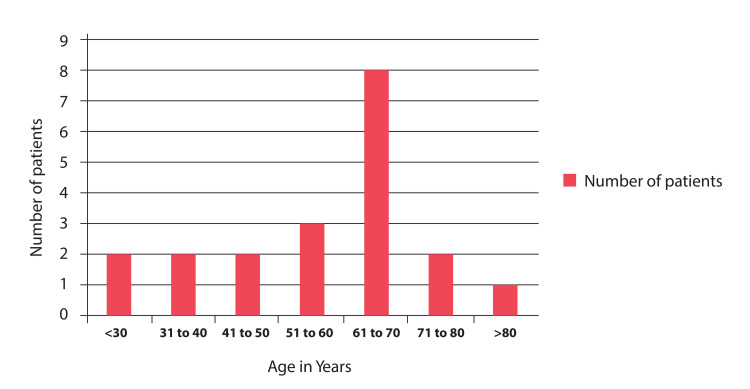
Age groups

The mean age of the patients at the time of presentation was 57 years, with a standard deviation of ±18.1. The age range varied from 17 to 80 years. Among the patients, 55% (12 individuals) were females, as indicated in Figure 1.

The most frequently reported symptom among the patients was dyspnea, which was experienced by 40% of the cases (n=8). Peripheral embolism, including cerebrovascular accidents and acute monocular vision loss, was the second most common symptom, observed in 20% of the patients (n=4). Other symptoms included chest pain (n=2, 10%), tachyarrhythmia (n=1, 5%), and syncope (n=1, 5%).

Among the patients with dyspnea (n=8), 12.5% (one patient) had severe congestive heart failure with New York Heart Association (NYHA) class IV, 50% (four patients) were markedly limited NYHA class III, and 38.5% (three patients) reported mild shortness of breath during ordinary activity or NYHA class II. Four patients (20%) presented with embolic manifestations, including two cases of cerebral embolism and two cases of acute monocular visual loss. Two patients (10%) presented with chest pain, one of whom had a myocardial infarction and subsequently underwent coronary artery bypass grafting (CABG). One patient (5%) had palpitations, and atrial fibrillation (AF) was detected on their electrocardiogram (ECG). Additionally, one patient (5%) presented with syncope, while two patients remained asymptomatic. None of the patients had a family history of myxoma.

**Figure 2:**
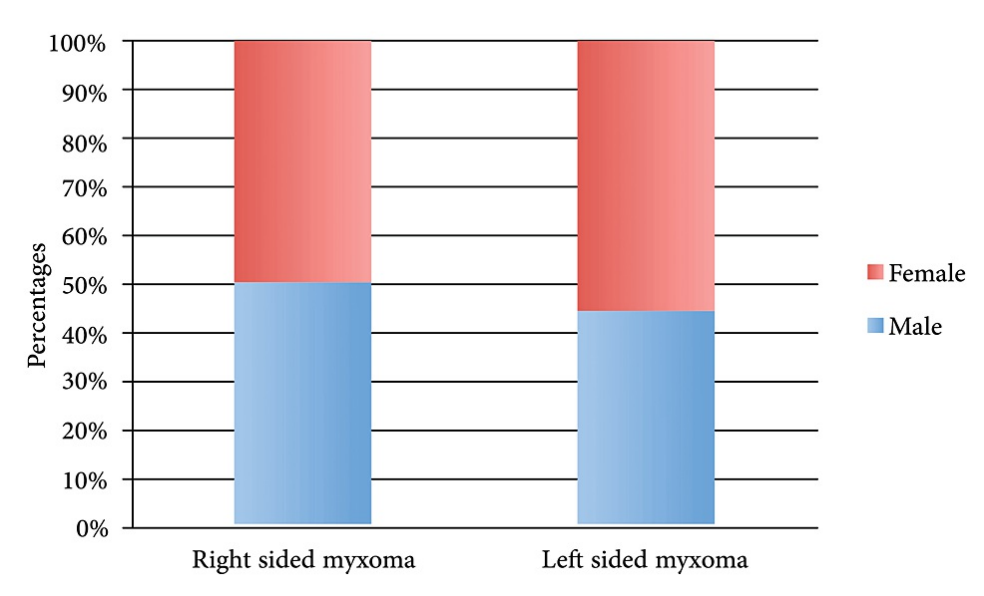
Gender and site distribution of atrial myxoma

The study describes the characteristics of myxoma, including its size and location. The largest diameter observed was 5.1 cm, with a standard deviation of ±1.7. Myxomas were predominantly found in the left atrium in 80% of cases (n=16), while 16% (n=4) presented in the right atrium. The majority of myxomas were attached to the atrial septum and fossa ovalis (Figure 2).

The operative features and surgical outcomes are presented in Table 2. All patients underwent complete resection of the tumour, with no residual tumour remaining. Median sternotomy and cardiopulmonary bypass were performed for all patients. The mean aortic cross-clamping time was 65 minutes (±37.8), and the mean time on cardiopulmonary bypass was 80 minutes (±39.2). Additional procedures were undertaken in some cases, including coronary artery bypass graft (CABG), mitral valve replacement (MVR), mitral valve repair (MVr), aortic valve replacement (AVR), tricuspid valve repair (TVr), and atrial septal defect (ASD) closure.

**Table 2:** Operative features and clinical outcomes CABG: coronary artery bypass graft; ASD: atrial septal defect

Operative and Clinical Outcomes	Values
Cardiopulmonary bypass time (minutes), mean ± SD	80 ± 39.2
Cross clamp time (minutes), mean ± SD	65 ± 37.8
Other Procedures	
CABG, n (%)	3 (15%)
Mitral valve replacement, n (%)	2 (10%)
Mitral valve repair, n (%)	2 (10%)
Tricuspid valve repair, n (%)	1 (5%)
Aortic valve replacement, n (%)	1 (5%)
ASD closure, n (%)	5 (25%)
Peri-Operative Complications	
None, n (%)	16 (80%)
Supraventricular arrhythmia, n (%)	2 (10%)
Pneumonia/pleural effusion, n (%)	2 (10%)
Acute blood loss necessitating blood transfusion, n (%)	3 (15%)
Demise, n	0
Intensive care stay (days), mean ± SD	1.8 ± 1.6
Hospital stay (days), mean ± SD	10 ± 5.8
Recurrence, n (%)	1 (5%)
All-cause mortality, n	0

Postoperatively, atrial fibrillation (AF) was the most common arrhythmia observed, occurring in 10% of patients (n=2). Pleural effusion was reported in 10% of cases (n=2), and acute blood loss necessitating blood transfusion occurred in 15% of cases (n=3). There were no documented in-hospital mortalities. The mean stay in the intensive care unit (ICU) was 1.8 days (±1.6), and the mean hospital stay was 10 days (±5.8).

**Figure 3:**
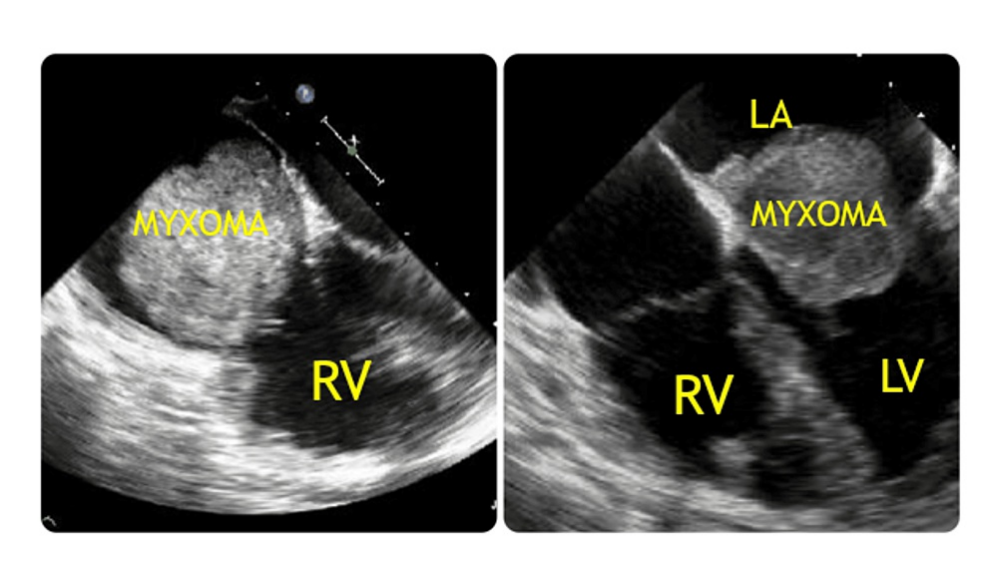
TEE studies showing right and left atrial myxoma, attached to inter-atrial septum. LA: left atrium; LV: left ventricle; RV: right ventricle; TEE: transesophageal echocardiography

During the follow-up period, one patient (5%) experienced a recurrence of myxoma at the same site (right atrium), three years after the initial diagnosis and resection. Histopathological verification was conducted for all cases. Figure 3 shows TEE of the right and left atrial myxomas attached to inter-atrial septum. 

## Discussion

Primary cardiac tumours have an incidence of 0.00138-0.03%, with 80% of them being benign [8]. Elbardissi et al. investigated 323 Mayo Clinic patients treated between 1957 and 2006 and discovered that 50% of cardiac tumours were myxomas and 26% were papillary fibroelastomas [9]. Other studies indicate that myxomas account for less than 75% of cases [10]. The most common benign primary heart tumours in adults are CMs, which account for 50-85% [11]. According to the World Health Organisation (WHO), a CM is a tumour composed of stellate to plump mesenchymal cells that are cytologically bland and embedded in a myxoid stroma. Females are more likely to develop atrial myxomas, which peak between the fourth and sixth decades of life. When viewed up close, atrial myxomas have a pedunculated appearance and have a soft texture. The myxoma's diameter ranges from 1 cm to 15 cm, and its weight ranges from 15 gm to 180 gm. Smooth, villous, or friable tumour surfaces are possible tumour presentations. When it comes to myxomas, the villous and friable ones are more likely to cause emboli, while the smooth ones are larger and more likely to cause obstruction. There are times when atrial myxomas release vascular endothelial growth factor (VEGF), which helps blood vessels grow. These tumours also release other cytokines and growth factors that lead to general symptoms like fever, tiredness, loss of appetite, weight loss, and a high sedimentation rate [12].

According to research, the female-to-male ratios for left and right atrial myxomas are 2.05:1 and 0.75:1, respectively [12], and in other published studies, the average age of the participants was between 50 and 55 years old [13]. In our study, we found that 55% of patients were female, with an average age of 57 ± 18.1 when they underwent surgery. According to the literature, the most common location (75% of myxomas) was the left atrium (80% in the current study), 15-20% in the right atrium (20% in the current study), and only 3-4% in the ventricles. The most common site of attachment is almost always in the region of the limbus of the fossa ovalis. On rare occasions, myxomas are seen on the posterior left atrial wall. In the present study, the site of attachments is consistent with international data [14].

Patients with CM may exhibit at least one or more of the tetrad symptoms: (i) arrhythmia, (ii) obstruction of intracardiac flow, (iii) systemic thromboembolism (cerebral and peripheral), and (iv) constitutional symptoms [5]. The symptoms of CM are more pronounced when they are left-sided and >5 cm in diameter. Siminelakis et al. observed that 46.2% of patients experienced embolism, 16% had dyspnea, 8% had constitutional symptoms, 8% had syncope, and 23.1% had myxoma discovered accidentally [15]. In a bigger study, 68% of patients experienced dyspnea and 40% reported embolism [16]. According to Elbardissi et al., embolic events occur at a rate of 24.8%, with left atrial and aortic valve tumours being more likely to be the source of embolic events [17]. A small volume and the absence of mitral regurgitation may be associated with an increased risk of embolic events. According to Perek et al., 62.5% of patients have dyspnea, 26.6% have embolism, 34.4% have systemic disease, and 17.2% are asymptomatic [18]. In our study 40% of patients presented with dyspnea, 20% with systemic embolism, 10% with chest pain, 5% with arrhythmias, and 5% with syncope.

TTE is the diagnosis modality of choice which is most used for the diagnosis giving the tumour location, shape, size, attachment, and mobility of the atrial mass, as well as the tumor's size, which can restrict circulation and serve as an emboli source. TEE is superior and more sensitive (sensitivity 100%) as compared to TTE. Other modalities like T1 - T1-weighted cardiac magnetic resonance imaging (CMRI), computed tomography (CT), and positron emission tomography (PET) can be used with lesser sensitivity [19].

Because of the higher risk of systemic embolization, cardiovascular problems, and sudden cardiac death, simple tumour excision by median sternotomy and cardiopulmonary bypass is the gold standard treatment for atrial myxoma once a preliminary diagnosis has been determined based on imaging investigations. Using the heart-lung machine as a cardiopulmonary bypass prevents tumour material from dislodging and generating systemic embolization. When compared to traditional open surgery, minimally invasive robotic surgery resulted in a shorter hospital stay with no discernible impact on quality of life afterwards. Endoscopic robotic excision of a left atrial myxoma has also been reported with a positive outcome [19].

Approximately 10-40% of patients experience postoperative atrial arrhythmias and atrioventricular conduction abnormalities, whereas 3% of patients experience postsurgical neurologic problems, with 5% requiring bleeding exploration [20]. In the present study, postsurgical AF was noted in 10% of cases, which was consistent with international data. The mean time in ICU was 1.8 days, which is almost in line with most of the literature. The time between surgical procedure and discharge (10 days) is consistent with reported international data of 8-10 days. There was no in-hospital death reported, which is low compared with other studies (3-9%) [16]. The prognosis for the patient is excellent in the long run. The risk of recurrence after surgery was estimated at 2-5%, with the most prevalent causes being a family history of the disease, tumours of unrecognized multicentric origin, insufficient tumour excision, intraoperative tumour cell spread, growth from a secondary focus, and de novo proliferation of pre-neoplastic or reserve cells in the endocardium. Early detection of recurring tumours can be achieved using biannual echocardiograms [20]. In the present study, only one patient (5%) had recurrence, which is consistent with published international data.

Limitations

The main constraint of this study on the epidemiology of CM in the Kingdom of Bahrain is the limited sample size, consisting of just 20 individuals. However, given the rarity of CM, we have employed our most effective methodology to report the findings. We have taken up all of the cardiac surgical cases for the entire Kingdom of Bahrain, which is noteworthy because there is only one cardiac centre in the country. Additionally, a small sample may hinder the ability to detect rare or subtle patterns in the prevalence, risk factors, or outcomes associated with CM in this specific region. Larger and more diverse participant groups would provide a more robust foundation for drawing meaningful conclusions and implications.

## Conclusions

This study provides a comprehensive analysis of the 10-year experience of CM in Bahrain. The findings contribute to existing knowledge on the prevalence and characteristics of this rare cardiac tumour. Understanding the epidemiology, clinical presentation, and management of CM is crucial for accurate diagnosis and effective treatment. The insights gained from this study can inform medical professionals in Bahrain and beyond, ultimately leading to improved patient care and outcomes in the management of CM.
